# Characterization and Functional Analysis of Fads Reveals Δ5 Desaturation Activity during Long-Chain Polyunsaturated Fatty Acid Biosynthesis in Dwarf Surf Clam *Mulinia lateralis*

**DOI:** 10.3390/genes15030365

**Published:** 2024-03-15

**Authors:** Tianhao Teng, Zhenghua Zheng, Wenqian Jiao, Na Liu, Ao Wang, Mengjiao Liu, Le Xie, Zujing Yang, Jingjie Hu, Zhenmin Bao

**Affiliations:** 1 MOE Key Laboratory of Marine Genetics and Breeding, College of Marine Life Sciences, Ocean University of China, Qingdao 266003, China; tengtianhao1998@163.com (T.T.); zhengzhenghua818@126.com (Z.Z.); deao_wang@hotmail.com (A.W.);; 2 Key Laboratory of Tropical Aquatic Germplasm of Hainan Province, Sanya Oceanographic Institution, Ocean University of China, Sanya 572000, China

**Keywords:** *Mulinia lateralis*, fatty acid desaturase, LC-PUFA, marine mollusks, microalgae

## Abstract

Fatty acid desaturases (Fads), as key enzymes in the biosynthesis of long-chain polyunsaturated fatty acids (LC-PUFAs), catalyze the desaturation between defined carbons of fatty acyl chains and control the degree of unsaturation of fatty acids. In the present study, two Fads genes, designated *MulFadsA* and *MulFadsB*, were identified from the genome of the dwarf surf clam *Mulinia lateralis* (Mollusca, Mactridae), and their spatiotemporal expression was examined. MulFadsA and MulFadsB contained the corresponding conserved functional domains and clustered closely with their respective orthologs from other mollusks. Both genes were expressed in the developmental stages and all tested adult tissues of *M. lateralis*, with *MulFadsA* exhibiting significantly higher expression levels in adult tissues than *MulFadsB*. Subsequently, the effects of dietary microalgae on *Fads* expressions in the dwarf surf clam were investigated by feeding clams with two types of unialgal diets varying in fatty acid content, i.e., *Chlorella pyrenoidosa* (Cp) and *Platymonas helgolandica* (Ph). The results show that the expressions of *MulFads* were significantly upregulated among adult tissues in the Cp group compared with those in the Ph group. In addition, we observed the desaturation activity of MulFadsA via heterologous expression in yeasts, revealing Δ5 desaturation activity toward PUFA substrates. Taken together, these results provide a novel perspective on *M. lateralis* LC-PUFA biosynthesis, expanding our understanding of fatty acid synthesis in marine mollusks.

## 1. Introduction

Long-chain polyunsaturated fatty acids (LC-PUFAs), such as n-3 eicosapentaenoic acid (EPA), docosahexaenoic acid (DHA), and n-6 arachidonic acid (ARA), are essential for human health [1,2]. LC-PUFAs play an important role in blood clotting, immune system regulation, neuro-transmitters, cholesterol metabolism, and the structure of membrane phospholipids in the brain and the retina [3]. As humans cannot synthesize the precursor fatty acids linoleic acid (LA, 18:2n-6) and α-linolenic acid (ALA, 18:3n-3), which are required for the synthesis of EPA, DHA, and ARA, these must be obtained through the diet, primarily from seafood [4]. Fish, especially oily fish from cold water, such as cod, tuna, and mackerel, are excellent sources of long-chain n-3 polyunsaturated fatty acids (n-3 PUFAs), predominantly EPA and DHA. Marine fish are a better source of n-3 essential fatty acid (EFA), while freshwater fish are a good source of n-6 EFA [5,6]. However, overfishing has significantly reduced the availability of fish as EPA, DHA, and ARA sources, necessitating the exploration of alternative sources. Marine mollusks, mainly bivalves, are emerging as a potential dietary resource, as they are rich in LC-PUFAs [7] and have the ability to endogenously biosynthesize LC-PUFAs [8,9].

LC-PUFA biosynthesis in vertebrates, which involves a series of desaturation and elongation reactions, is mediated primarily via fatty acid desaturases (Fads) and elongases (Elovls); this has been extensively studied [10,11]. The complement and catalytic activities of Fads and Elovls, especially for Fads, determine the LC-PUFA biosynthesis ability of organisms. In marine mollusks, Fads with Δ5 desaturation activity were first identified from the cephalopod *Octopus vulgaris*, which was a key step for the biosynthesis of LC-PUFAs [12]. Subsequently, similar Δ5 Fads were also characterized in other mollusks, including abalone (*Haliotis discus hannai*) [13] and razor clam (*Sinonovacula constricta* [14]. Furthermore, Δ8 Fads and Δ6 Fads were subsequently identified from the noble scallop (*Chlamys nobilis*) [15] and common cuttlefish (*Sepia officinalis*) [14], respectively. All these studies have demonstrated that mollusks have different Fads with various desaturation abilities. Additionally, it has been found that the variable marine environment, particularly the availability of LC-PUFA-rich diets, has a great effect on the fatty acid profiles and biosynthetic potential of marine mollusks [16,17,18]. Specifically, the expression of genes involved in the LC-PUFA synthesis of marine mollusks is affected by microalgae diets, as the fatty acid contents of microalgae vary. For example, feeding with *Chlorella* sp. can significantly upregulate the *Fads* gene expression of the manila clam (*Ruditapes philippinarum*) [19].

The dwarf surf clam (*Mulinia lateralis*, 1822), a small filter-feeding bivalve mollusk, is predominantly distributed in estuarine and intertidal zones along the Gulf of Mexico, the West Indies, and the Atlantic coast [20]. With a short generation cycle, rapid reproduction, strong environmental adaptability, and ease of artificial breeding, the dwarf surf clam is a potential model organism for bivalves [21,22]. At present, we have successfully established an artificial breeding system for *M. lateralis*, and investigated the effects of different microalgae diets on its development and growth performance [23]. The microalgae species with which it is typically fed, including *Chlorella pyrenoidesa* (Cp), *Platymonas helgolandica* (Ph), and other several types of microalgae, can be taken up by spats of *M. lateralis.* There are significant differences in the growth of spat fed with these microalgae. It has also been demonstrated that differences in the nutrient contents of these microalgae result in differences in the growth of spats [24]. Recently, we have also found that the varied fatty acid contents of the microalgae diets lead to differences in the fatty acid composition of adult tissues in *M. lateralis*. However, the composition and function of *Fads* genes involved in LC-PUFA biosynthesis in *M. lateralis* and the influence of diet on their expression are still unknown. Moreover, whether *M. lateralis*, as a potential model organism for bivalves, has the biosynthesis activity of LC-PUFAs, especially with respect to EPA, DHA, and ARA, is still unknown.

Therefore, in this study, we identified the *Fads* of *M. lateralis* and analyzed the differential expression patterns of *Fads* among various tissues and developmental stages. We also investigated the effects of different unialgal diets on *Fads* expression in *M. lateralis*. Moreover, we conducted heterologous expression of Fads in yeast to explore their desaturation activity. These findings will contribute to a better understanding of *Fads* genes and LC-PUFA synthesis in *M. lateralis*, and may potentially be useful for other mollusks.

## 2. Materials and Methods

### 2.1. Gene Identification and Sequence Analysis of Fads in M. lateralis

To identify the Fads genes of *M. lateralis*, the genome database of *M. lateralis* was searched using 28 Fads amino acid sequences from 11 representative species, including mammals (*Homo sapiens* and *Mus musculus*), fish (*Danino rerio*, *Salmo salar*, and *Oncorhynchus mykiss*), and other mollusks (*Mercenaria mercenaria*, *R. philippinarum*, *S. constricta*, *Crassostrea gigas*, *Patinopecten yessoensis*, *Chlamys farreri*, *C. nobilis*, *Aplysia californica*, *H. discus hannai*, *O. vulgaris*, and *S. officinalis*). This search was conducted via the TBLASTN algorithm with an E-value cutoff of 1E-05. The orthologous Fads amino acid sequences were downloaded from the NCBI database. Then, the hit target sequences were used to search against a transcriptome database of *M. lateralis* and sequences that obtained hits with an E-value threshold of 1E-05 were regarded as candidate Fads. To validate these candidates, a BLASTP search against the NCBI non-redundant protein sequence database was performed. To guarantee the completeness and accuracy of the Fads sequences, the ORF finder tool was utilized to predict the nucleotide sequence, and the amino acid sequence was submitted to the SMART database for Fads domain verification. A genetic structure diagram of Fads was generated using the dwarf surf clams’ general feature format (GFF) gene annotation file. A conserved domain analysis of *M. lateralis* Fads sequences was conducted using DNAMAN7.0 [25].

### 2.2. Phylogenetic Analysis of Fads

Fads protein sequences from *M. lateralis* and representative mammals, fish, and other mollusks (mentioned in Section 2.1) were chosen for phylogenetic analysis. ClustalW2.1 was employed for multiple Fads protein sequence alignment, and MEGA7.0 [26] was used for phylogenetic analysis using the maximum likelihood (ML) method with a bootstrap value of 5000 to ensure the reliability of the constructed evolutionary tree.

### 2.3. Spatiotemporal Expression of Fads in M. lateralis

According to RNA-seq datasets of *M. lateralis* obtained in our laboratory, the expression profiles of *MulFads* were analyzed. The expression level was described by transcripts per kilobase per million mapped reads (TPM) from the RNA-seq datasets, including 11 developmental stages (egg, zygote, 2–4 cells, 8–16 cells, multicellular, blastula, gastrula, trochophore, D-shaped larvae, umbo larvae, and juvenile) and 4 adult tissues (mantle, foot, digestive gland, and gonad). The samples representing various developmental stages were obtained through artificial fertilization from a cohort of fifty mature adults, followed by larval cultivation. Additionally, various tissues from three distinct adult individuals were selected for RNA extraction and subsequent transcriptomic sequencing.

### 2.4. Expression Analysis of Fads in M. lateralis under Two Unialgal Diets

The transcriptomic databases of *M. lateralis* in response to two unialgal diets, *C. pyrenoidesa* (Cp) and *P. helgolandica* (Ph), were constructed (unpublished data) and the expression levels of *MulFads* were detected. Specifically, the feeding trials were conducted with three-month-old *M. lateralis* over a period of two weeks. For each trial, 20 individuals were housed in an aquarium tank measuring 30 cm × 40 cm × 35 cm and were fed with CP or Ph twice daily, at 10:00 a.m. and 10:00 p.m. The water temperature in all tanks was consistently maintained at 22 ± 1 °C, with a salinity level of 27 ± 1 ppt. After the two-week period, three individuals from each feeding group were randomly selected for sample collection. Four types of tissues (mantle, foot, digestive gland, and gonad) were harvested for RNA extraction and subsequent transcriptomic sequencing. The expression levels of *MulFads* in response to Cp and Ph diets were described by transcripts per kilobase per million mapped reads (TPM) from these RNA-seq datasets.

### 2.5. Functional Characterization via Heterologous Expressions of M. lateralis Fads Open Reading Frame in Yeasts

The *MulFadsA* gene was chosen for constructing the recombinant plasmid pYES2-*MulFadsA* using the pYES2.0 yeast expression vector (Coolaber, Beijing, China), which was synthesized by Sangon Biotech Shanghai Co., Ltd (Sangon Biotech, Shanhai, China). This recombinant plasmid was transformed into *Saccharomyces cerevisiae* INVSc1 competent cells (Coolaber, Beijing, China), and yeast harboring pYES2-*MulFadsA* was selected on SCMM-uracil minimal medium (Coolaber, Beijing, China). The selected yeast was subsequently cultivated in SD-U liquid medium (Coolaber, Beijing, China) until an OD400 of 1.0 was reached, followed by concentration and resuspension in 2 mL SG-U induction medium (Coolaber, Beijing, China). The concentrated yeast was added to 100 mL of SG-U induction medium to adjust the OD400 to 0.4.

To investigate the desaturation activity of Fads in the synthesis of LC-PUFAs in the clam, yeast transformed with pYES2-*MulFadsA* was cultured with exogenous supplementation 18:3n-3 (Aladdin, Shanghai, China) and 18:2n-6 (Solarbio, Beijing, China) for the Δ6 desaturase substrate fatty acids, 20:3n-3 (Aladdin, Shanghai, China) for the Δ8 desaturase substrate fatty acids, and 20:3n-6 (Aladdin, Shanghai, China) for the Δ5 desaturase substrate fatty acids, at a final concentration of 0.5 mM. The yeast culture was induced for 72 h, after which the yeast cells were centrifuged and freeze-dried for subsequent fatty acid analysis.

### 2.6. Fatty Acid Analysis Using GC-MS

The analysis of fatty acids in yeast powder was performed by Qingdao Kechuang Quality Testing Co., Ltd. (Qingdao, China) using a Thermo (race1310 ISQ, Thermo, Waltham, MA, USA) gas chromatograph. The conversion rate of fatty acid substrate to desaturated product was determined using the equation [product area/(product area + substrate area)] × 100%.

### 2.7. Statistical Analyses

Statistical analyses were conducted using SPSS 22.0 software (SPSS, Inc., Chicago, IL, USA). Data were analyzed with one-way ANOVA and Newman–Keuls tests. A *p*-value of <0.05 was deemed statistically significant.

## 3. Results

### 3.1. Gene Identification and Sequence Analysis of Fads in M. lateralis

**Figure 1:**
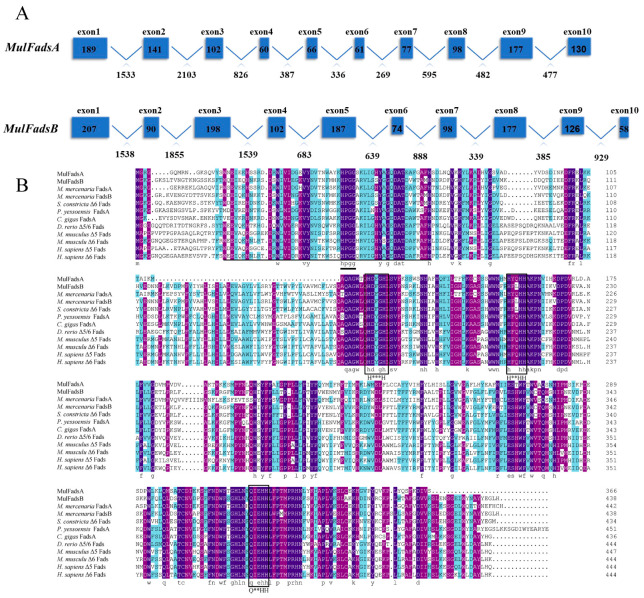
Sequence analysis of Fads. (**A**) Gene structure of the *MulFadsA* and *MulFadsB* of *M. lateralis*. The blue boxes and polylines indicate the exons and introns, respectively. The numbers in the boxes and under the line indicate the lengths of exons and introns, respectively. (**B**) Multiple sequence alignment of Fads sequence in *M. lateralis*. The cytochrome-b5 like domain is underlined with a solid line, the heme-binding motif of HPGG is highlighted with a short bold line, and the three histidine boxes are denoted with frames. An asterisk indicates any amino acid in the domain.

Two *Fads* genes were identified based on the transcriptome and genome of *M. lateralis*; these were named *MulFadsA* and *MulFadsB*. As shown in Table 1, the cDNA lengths of *MulFadsA* and *MulFadsB* were 1175 bp and 4510 bp, encoding 366 amino acids and 438 amino acids, respectively. Both *MulFadsA* and *MulFadsB* were composed of 10 exons and 9 introns (Figure 1A). Multiple sequence alignment of Fads protein sequences from *M. lateralis* and other species revealed that the aligned sequences encompass three conserved histidine-rich motifs (H***H, H**HH, and Q**HH), a putative cytochrome b5-like domain, and a conserved N-terminal heme-binding domain (HPGG) (Figure 1B). Meanwhile, the deduced amino acid sequences of *MulFadsA* and *MulFadsB* exhibited 63.76% homology to Fads of the hard clam (*M. mercenaria*), razor clam (*S. constricta*), Pacific oyster (*C. gigas*), and Yesso scallop (*P. yessoensis*).

### 3.2. Phylogenetic Analysis

**Figure 2:**
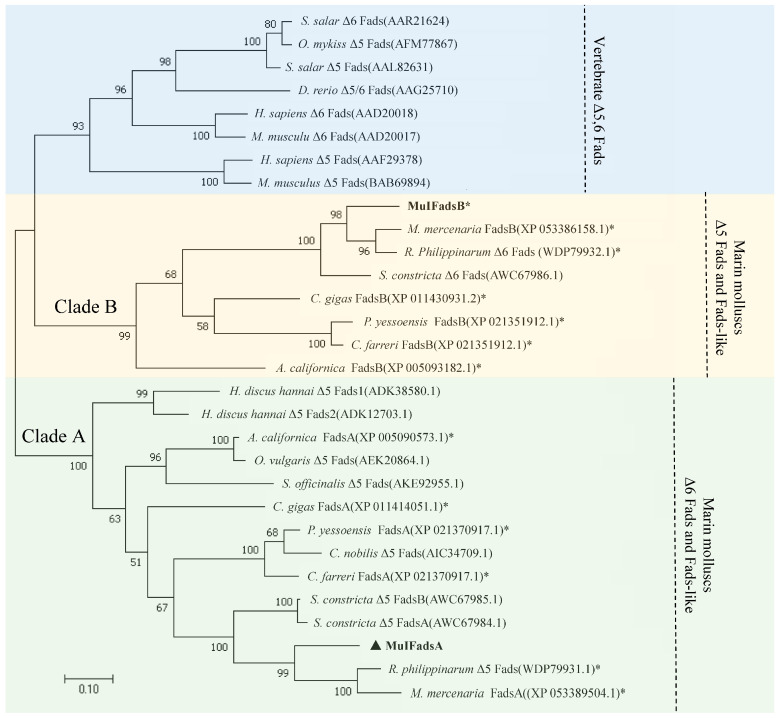
Phylogenetic tree of Fads proteins from *M. lateralis* and other representative species. The tree was constructed using MEGA7.0 via the maximum-likelihood (ML) method with a bootstrap value of 2000. The numbers represent the frequencies with which the tree topology presented was replicated. The scale bar indicates a branch length of 0.05. An asterisk indicates Fads sequences of *M. mercenaria*, *R. philippinarum*, *C. gigas*, *P. yessoensis*, *C. farreri*, and *A. californica* that have not been functionally characterized. The black solid triangle represents the sequence validated in this experiment.

An ML phylogenetic tree was constructed using the amino acid sequences of MulFads and Fads members from other species. All the mollusk front-end desaturases were classified into two distinct clades identified as clade A and clade B (Figure 2) according to the nomenclature proposed by Surm et al. [27,28] and Ramos-Llorens et al. [29]. Specifically, clade A contained all functionally characterized Δ5 Fads and Fads-like sequences of other mollusks (not functionally characterized, denoted with *). MulFadsA, together with another two Fads from *R. philippinarum* and *M. mercenaria*, were clustered into this clade. Clade B contained more Fads-like sequences of mollusks that were not functionally characterized, except for Δ6 Fads sequences functionally characterized from *S. constricta*, and MulFadsB were clustered into this clade.

### 3.3. Spatiotemporal Expression of MulFads

**Figure 3:**
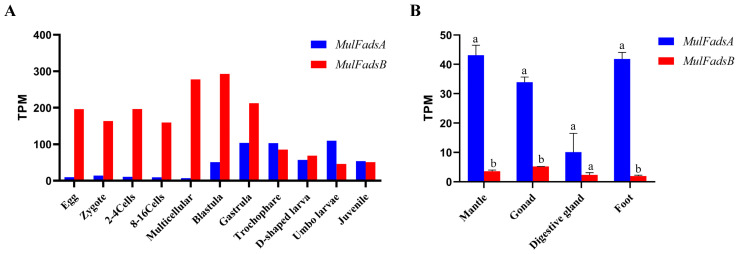
Expression pattern of *MulFads* from *M. lateralis*. (**A**) Expressions of *MulFadsA* and *MulFadsB* in *M. lateralis* at 11 developmental stages based on the transcripts per kilobase per million mapped reads (TPM). (**B**) Expressions of *MulFadsA* and *MulFadsB* in *M. lateralis* adult tissues. SPSS28.0 was used for one-way ANOVA analysis of the data, and the Duncan method was used to test the significance of the difference between the values of each group. Different letters indicate significant differences among experimental groups (*p* < 0.05).

RNA-seq datasets for different developmental stages and adult tissues of *M. lateralis* were analyzed to detect the spatiotemporal expression patterns of *MulFadsA* and *MulFadsB* genes (Figure 3). Specifically, the expression of *MulFadsA* in the early developmental periods was low, with TPM below X, and then gradually increased from blastula stage (Figure 3A). *MulFadsB* showed the opposite expression pattern, with high expression levels in eggs and during early developmental periods and low expression levels from the trochophore stage (Figure 3A). During complete developmental stages, *MulFadsA* showed lower expression levels compared with *MulFadsB*. In adult tissues of *M. lateralis*, the overall expression level of *MulFadsA* was higher than that of *MulFadsB*. *MulFadsA* had significantly higher expression levels in the mantle, gonad, and foot compared to the digestive gland (Figure 3B). The expression level of *MulFadsB* was highest in the gonad, followed by the mantle, digestive gland, and foot (Figure 3B).

### 3.4. Effects of Different Unialgal Diets on Fads Gene Expression in M. lateralis

**Figure 4:**
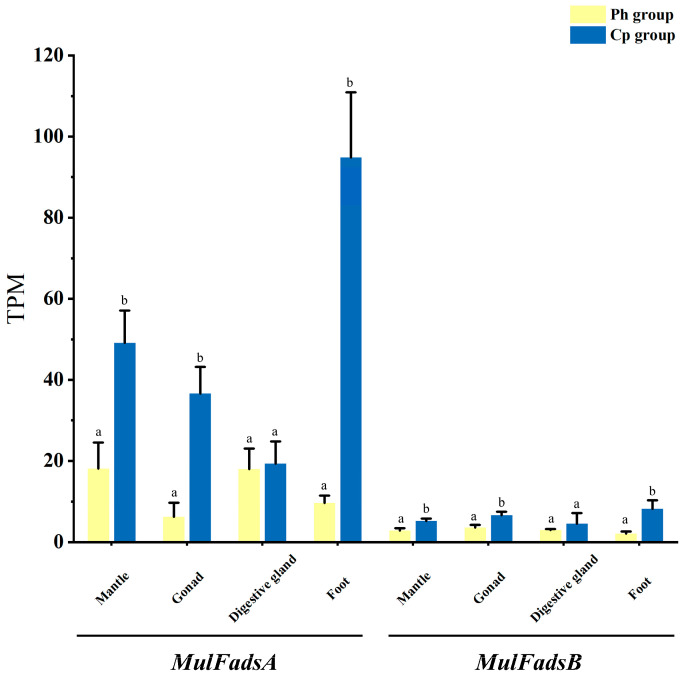
Gene expression of *M. lateralis Fads* under different dietary conditions in adult tissues. Different letters indicate significant differences among experimental groups (*p* < 0.05, Tukey’s test). Cp: *C. pyrenoidesa*; Ph: *P. helgolandica*.

To examine the expression patterns of *MulFads* genes in response to different unialgal diets, RNA-seq datasets of four tissues from *M. lateralis* under two types of unialgal diets, including *C. pyrenoidosa* (Cp) and *P. helgolandica* (Ph) with a distinct LC-PUFA composition [23], were used for analysis. According to our previous research [24] and a recent study (unpublished), the Cp diet has a positive effect on the growth and fatty acid composition of *M. lateralis* as compared to the Ph diet. The feeding effects of the two microalgae diets on the expressions of *MulFads* are shown in Figure 4. Specifically, *MulFadsA* expression levels were significantly higher in the mantle, gonad, and foot of the Cp group compared to those in the Ph group, while no significant difference was found for the digestive gland between the two groups. *MulFadsB* showed a similar expression pattern between the two groups but with a notably lower expression level than *MulFadsA* (Figure 4).

### 3.5. Functional Characterization of M. lateralis Fads

**Figure 5:**
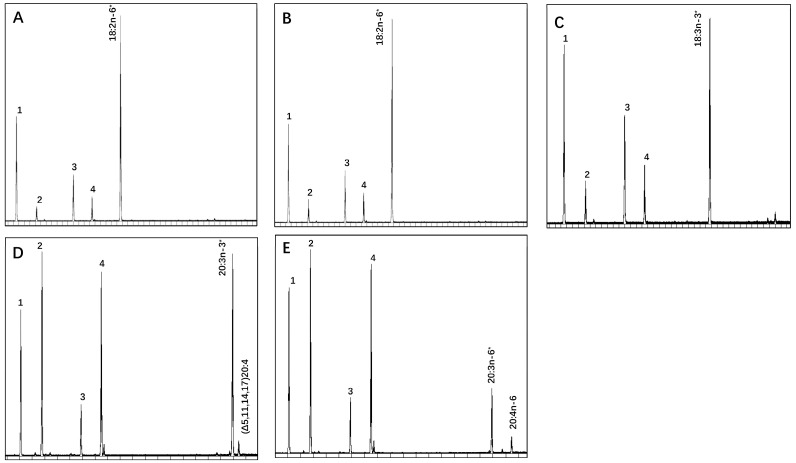
Functional characterization of *MulFadsA*. Yeasts transformed with pYES2 alone (**A**) and pYES2-*MulFadsA* (**B**–**E**) were cultured in the presence of FA substrates (highlighted with *). Peaks 1–4 represent the main endogenous FAs of *S. cerevisiae*, namely C16:0 (1), C16:1n-7 (2), C18:0 (3), and C18:1n-9 (4). The only additional peak was identified as C20:4n-6 (**E**). The vertical axis represents the flame ionization detector (FID) response, and the horizontal axis represents the retention time.

Following the identification and expression analysis, we selected the *MulFadsA* gene, which exhibited high expression levels and significant influence from algal dietary conditions, for subsequent functional validation experiments. Yeasts with empty pYES2 only contained endogenous FAs, including C16:0, C16:1n-7, C18:0, and C18:1n-9 (peaks 1–4) (Figure 5A), and exogenously added PUFAs (denoted with *) (Figure 5B–E). The results are consistent with the lack of PUFA desaturase activity in *S. cerevisiae* [30,31,32]. Yeast transformed with pYES2-*MulFadsA* recombinant plasmid was grown in the presence of Δ6-(18:2n-6 and 18:3n-3), Δ8-(20:3n-3), and Δ5-desaturation (20:3n-6) substrates (Figure 5B–E, respectively). The results indicate that only exogenously added 20:3n-6 was converted into 20:4n-6 (Figure 5E), indicating that MulFadsA exhibited Δ5 desaturation activity. According to the conversion rate, as presented in Table 2, 26% of C20:3n-6 was desaturated to C20:4n-6 by MulFadsA.

## 4. Discussion

The *Fads* gene encodes the crucial rate-limiting enzyme in the synthesis of LC-PUFAs through desaturation, which is a front-end desaturase [33]. Typical front-end desaturases of animals in the biosynthesis of LC-PUFAs include Δ4, Δ5, Δ6, and Δ8 fatty acid desaturases. Various *Fads* genes demonstrating a range of desaturation activity, such as Δ4, Δ5, Δ6, Δ8, Δ5/6, and Δ6/8, have been identified in mammals and teleost fish [30,34,35,36,37,38]. In addition, a series of studies have demonstrated that Fads with ∆5, Δ6, or Δ8 activity appear to be widely distributed among mollusks, including cephalopods [12,39], gastropods [13], and bivalves [14,15,19,40]. Generally, it is accepted that mollusks possess the ability to facilitate PUFA biosynthesis [41]. However, such capability appears to vary among species depending on the complement of rate-limiting enzymes involved in this process. The expanding availability of genomic data from various invertebrate taxa now enables detailed investigations of the LC-PUFA biosynthetic pathways at a molecular level, focusing on the characterization of crucial biosynthesizing enzymes. In our study, with the help of the whole genomic and transcriptomic database of *M. lateralis*, we systematically conducted the identification and phylogenetic analysis of two *MulFads* genes, examined their spatiotemporal expression profiles, and investigated their responses to two types of unialgal diets. Then, we determined the desaturation activity of MulFadsA enzymes via a yeast heterologous expression assay, which confirmed that Δ5 desaturation activity was characterized.

Two Fads genes, *MulFadsA* and *MulFadsB*, were successfully identified in the genome of *M. lateralis.* Both MulFadsA and MulFadsB showed high homology with Fads sequences from other representative vertebrates and invertebrates and typically have the structural characteristics of front-end desaturases, including a cytochrome b5-like domain, three typical histidine boxes (H***H, H**HH, and Q**HH), and a heme-binding motif, i.e., HPGG (Figure 1B), indicating the conserved functional domains of Fads during evolution [39] and the potential desaturation activity of *M. lateralis* Fads. In addition, the phylogenetic analysis revealed that MulFadsA was clustered together with Δ5 functionally characterized Fads from other marine mollusks, indicating the potential corresponding Δ5 Fad activity of MulFadsA (Figure 2). Meanwhile, MulFadsB was clustered together with Δ6 Fads from other marine mollusks (Figure 2), which is consistent with the phylogenetic results with respect to Fads from *R. philippinarum* [19] and other mollusks [27]. These results indicate that both Δ5 and Δ6 Fads might exist in *M. lateralis* and other marine mollusks [14,19].

Furthermore, spatial and temporal expression analyses of *MulFadsA* and *MulFadsB* revealed different expression trends across developmental stages and in adult tissues. During the developmental process of *M. lateralis*, *MulFadsB* was expressed in eggs and subsequently primarily expressed in the early embryonic developmental stages (Figure 3A). This result suggests that *MulFadsB* transcripts in the eggs and early larvae are maternally derived rather than endogenously expressed [42]. A similar maternal expression pattern of *Fads* has also been reported in *Danio rerio* [42], Common Carp *Cyprinus carpio* [38], and noble scallop *C. nobilis* [40]. While the *MulFadsA* transcript was low at the early developmental stage, it increased dramatically from blastula stages to juvenile stages, indicating that Δ5 Fads may be a critical enzyme in the biosynthesis of PUFAs that are needed in normal development during the early stages in mollusks [40] and other animals [42]. This result indicates that the maternal *MulFadsB* transcript mainly regulates the metabolism of fatty acids and participates in the synthesis of HUFAs in the early embryonic developmental stages. After the blastula stage, as the lower maternal *MulFadsB* transcripts and higher *MulFadsA* transcripts, both *MulFadsA* and *MulFadsB* are involved in the synthesis of PUFAs. Based on our preliminary observations in this study, we hypothesize that these two genes may be involved in LC-PUFA synthesis and may be functional at different stages of *M. lateralis* embryonic development.

The tissue distributions show that both *MulFadsA* and *MulFadsB* exhibited significantly high expression levels in the gonad, and particularly high expression of *MulFadsA* was found in the mantle and foot (Figure 3B). Similar results with respect to tissue distributions of Δ5 *Fads* have also been observed in other marine mollusks [12,14,43]. For example, *O. vulgaris Fads* exhibit higher expression in the digestive gland and gonad [12]. Δ5 *Fads* in *S. constricta* also exhibit high expression levels in the gonad [14]. The expressions of *MulFadsA* and *MulFadsB* in the digestive gland were lower than those in other organs, which may indicate that different tissues have different requirements for long-chain fatty acid metabolism. Furthermore, the expression of *MulFadsA* in most tissues was higher than that of *MulFadsB*; it is speculated that *MulFadsA* plays a stronger role and is more efficient in the process of HUFA synthesis than *MulFadsB* in adult *M. lateralis.*

To provide insights into the function of *MulFads* in response to a microalgae diet, the expression of *MulFads* was investigated in four adult tissues under two different unialgal diets. Multiple factors regulate *Fads* gene expression, with diet being a significant factor [44]. In general, in most tissues of *M. lateralis*, the expressions of *MulFadsA* and *MulFadsB* were significantly upregulated in the Cp group compared to those in the Ph group (Figure 4). Specifically, feeding with *C. pyrenoidosa* has a more pronounced effect on *MulFadsA* expression in the mantle, gonad, and foot. This is similar to the findings reported by Wu et al. [19] in respect of the *Fads* of *R. philippinarum*. The expression levels of *Fads* in *R. philippinarum* fed with *Chlorella* sp. were significantly higher compared to those fed with *Chaetoceros calcitrans* and *Isochrysis galbana*. This result may be related to the lower content of LC-PUFAs in *Chlorella* sp., especially EPA and ARA, compared to *P. helgolandica* [40], resulting in upregulated expression of desaturase genes to promote the rapid synthesis of LC-PUFAs to meet the needs of growth and development. Similar findings have also been observed in crustaceans, such as *Cherax quadricarinatus* [45] and *Scylla paramamosain* [46], and in teleost fishes, such as *Siganus canaliculatus* [47], *S. salar* [48], and *O. mykiss* [49]; these studies showed that the expression levels of Fads were significantly upregulated in response to dietary DHA and EPA deficiency.

In terms of functional verification, our study shows that the expression products of *MulFadsA* can exhibit Δ5 Fads activity when using C20:3n-6 as the substrate, which is consistent with our phylogenetic results (Figure 2), indicating that MulFadsA clusters together with Δ5 Fads from other mollusks. Notably, we observed no desaturation when Δ6 substrates (C18:2n-6 and C18:3n-3) or Δ8 substrates (C20:3n-3) were used, indicating that this enzyme lacks Δ6 or Δ8 desaturase activities (refer to Figure 5 and Table 2). These findings imply that MulFadsA, with its Δ5 desaturase function, may play a crucial role in converting 20:3n-6 into ARA. In previous studies, *Octopus* Fads was the first enzyme to be shown to exhibit Δ5 desaturation activity in marine mollusks [12]. Subsequently, Δ5 Fads desaturation activity was verified in abalone (*H. discus hannai*) [13], noble scallop (*C. nobilis*) [40], common cuttlefish (*S. officinalis*) [39], razor clam (*S. constricta*) [14], and pearl oyster (*Pinctada fucata martensii*) [50]. In our study, the conversion rate of *M. lateralis* MulFadsA from C20:3n-6 to C20:4n-6 was 26% (Table 2), which was higher than that of *S. constricta* (10.41–13.71%) [14], *H. discus hannai* (about 15%) [13], and *C. nobilis* (about 17%) [40], but lower than that of *S. officinalis* [39] and *O. vulgaris* [12] (up to 39%). This may be due to the various desaturation activities among different mollusks. In addition, MulFadsB was clustered with Δ6 Fads that have functional properties in *S. constricta*, suggesting that MulFadsB may exhibit Δ6 desaturation activity (Figure 2); this needs to be further verified. Fads enzymes are pivotal in regulating the unsaturation levels of PUFAs, facilitating the initial steps of desaturation [10]. Specifically, Δ6 and Δ5 desaturases are essential for the synthesis of ARA from linoleic acid (LA, C18:2n-6), and eicosapentaenoic acid (EPA, C20:5n-3) from linolenic acid (LNA, C18:3n-3), respectively. Combined with the results of the functional verification of MulFadsA and the phylogenetic tree of MulFadsA and MulFadsB, it is speculated that MulFadsB may potentially exhibit Δ6 activity. As a result, it is speculated that *M. lateralis* might have the capacity to biosynthesize ARA and EPA/DHA from LA and ALA substrates, respectively. In future studies, we will select more diverse substrates to further verify the desaturation activity of MulFadsA and MulFadsB. We may also examine the activity of fatty acid elongases involved in LC-PUFA biosynthesis, thus allowing a better understanding of the LC-PUFA synthesis ability of *M. lateralis*.

## 5. Conclusions

In our comprehensive study on *M. lateralis* Fads, encompassing both *MulFadsA* and *MulFadsB*, we explored the expression patterns during embryonic development and across various tissues of *M. lateralis*. This investigation offers valuable insights into the mechanisms of LC-PUFA biosynthesis in mollusks. Our findings reveal that *MulFadsA* exhibits lower expression levels in the early stages of embryonic development, with an increase observed in the mid to late stages. Conversely, *MulFadsB* displays a contrasting pattern of expression. Additionally, when *M. lateralis* was fed with *C. pyrenoidosa*, a significant upregulation of *MulFads* expression was observed in the mantle, gonad, and foot, suggesting that the dietary absence of EPA and ARA triggers the upregulation of *MulFads*. The functional characterization of *MulFadsA* highlights the potential of the model bivalve *M. lateralis* to endogenously synthesize LC-PUFAs, and to at least convert C20:3(n-6) into ARA. This capability is not only pivotal in understanding the molecular evolution of the Fads family but also underscores the significance of LC-PUFA biosynthesis from fatty acid substrates in mollusks and other invertebrates. This study sheds light on the regulatory mechanisms of Fads in *M. lateralis*, contributing to our broader understanding of fatty acid metabolism in marine organisms.

## Figures and Tables

**Table 1:** Characteristics of the *Fads* genes in *M. lateralis*.

Gene Name	cDNA Length (bp)	ORF Length (bp)	Exon No.	Intron No.	Amino Acid No.	Molecular Weight (kDa)	Theoretical pI	α No.	Extended No.	Colis No.	Turn No.
*MulFadsA*	1175	1101	11	10	366	42.8	8.85	166	41	147	12
*MulFadsB*	4510	1317	11	10	438	51.38	8.45	207	51	163	17

**Table 2:** Functional characterization of *M. lateralis* MulFadsA in *S. cerevisiae*.

Substrate	Product	Conversion Rate	Activity
C18:2n-6	C18:3n-6	0%	Δ6
C18:2n-6	C18:3n-6	0%	Δ6
C18:3n-3	C18:4n-3	0%	Δ6
C20:3n-3	C20:4n-3	0%	Δ8
C20:3n-6	C20:4n-6	26%	Δ5

## Data Availability

The data presented in this study are available in the article.

## References

[1] Russo G.L. (2009). Dietary n-6 and n-3 polyunsaturated fatty acids: From biochemistry to clinical implications in cardiovascular prevention. Biochem. Pharmacol..

[2] Van Dael P. (2021). Role of n-3 long-chain polyunsaturated fatty acids in human nutrition and health: Review of recent studies and recommendations. Nutr. Res. Pract..

[3] Abedi E., Sahari M.A. (2014). Long-chain polyunsaturated fatty acid sources and evaluation of their nutritional and functional properties. Food Sci. Nutr..

[4] Williams C.M., Burdge G. (2006). Long-chain n-3 PUFA: Plant v. marine sources. Proc. Nutr. Soc..

[5] Ugoala C., Ndukwe G., Audu T. (2008). Comparison of fatty acids profile of some freshwater and marine fishes. Internet J. Food Saf..

[6] Pirestani S., Sahari M., Barzegar M., Nikoopour H. (2010). Lipid, cholesterol and fatty acid profile of some commercially important fish species from south Caspian Sea. J. Food Biochem..

[7] Joseph J.D. (1982). Lipid composition of marine and estuarine invertebrates. Part II: Mollusca. Prog. Lipid Res..

[8] Monroig Ó., Tocher D.R., Navarro J.C. (2013). Biosynthesis of polyunsaturated fatty acids in marine invertebrates: Recent advances in molecular mechanisms. Mar. Drugs.

[9] Castro L.F.C., Tocher D.R., Monroig O. (2016). Long-chain polyunsaturated fatty acid biosynthesis in chordates: Insights into the evolution of Fads and Elovl gene repertoire. Prog. Lipid Res..

[10] Cook H.W., McMaster C.R. (2002). Fatty acid desaturation and chain elongation in eukaryotes. New Comprehensive Biochemistry.

[11] Sprecher H. (2000). Metabolism of highly unsaturated n-3 and n-6 fatty acids. Biochim. Biophys. Acta.

[12] Monroig O., Navarro J.C., Dick J.R., Alemany F., Tocher D.R. (2012). Identification of a Δ5-like fatty acyl desaturase from the cephalopod *Octopus vulgaris* (Cuvier 1797) involved in the biosynthesis of essential fatty acids. Mar. Biotechnol..

[13] Li M., Mai K., He G., Ai Q., Zhang W., Xu W., Wang J., Liufu Z., Zhang Y., Zhou H. (2013). Characterization of two Δ5 fatty acyl desaturases in abalone (*Haliotis discus hannai Ino*). Aquaculture.

[14] Ran Z., Xu J., Liao K., Li S., Chen S., Yan X. (2018). Biosynthesis of polyunsaturated fatty acids in the razor clam *Sinonovacula constricta*: Characterization of Δ5 and Δ6 fatty acid desaturases. J. Agric. Food Chem..

[15] Liu H., Zhang H., Zheng H., Wang S., Guo Z., Zhang G. (2014). PUFA biosynthesis pathway in marine scallop *Chlamys nobilis* Reeve. J. Agric. Food Chem..

[16] Caers M., Coutteau P., Sorgeloos P. (1999). Dietary impact of algal and artificial diets, fed at different feeding rations, on the growth and fatty acid composition of *Tapes philippinarum* (L.) spat. Aquaculture.

[17] Lee M.C., Park J.C., Lee J.S. (2018). Effects of environmental stressors on lipid metabolism in aquatic invertebrates. Aquat. Toxicol..

[18] Zhukova N.V. (2019). Fatty Acids of Marine Mollusks: Impact of Diet, Bacterial Symbiosis and Biosynthetic Potential. Biomolecules.

[19] Wu K., Ran Z., Wu S., Xie H., Li Y., Liao K., Xu J., Yan X. (2023). Biosynthesis of LC-PUFA in *Ruditapes philippinarum*: Cloning and tissue distribution of Fad and Elovl, and effects of microalgae diets varied in LC-PUFA composition on their expressions and fatty acids profile of this bivalve. Front. Mar. Sci..

[20] Walker R.L., Tenore K.R. (1984). Growth and production of the dwarf surf clam *Mulinia lateralis* (Say 1822) in a Georgia estuary. Gulf Caribb. Res..

[21] Calabrese A. (1970). Reproductive cycle of the coot clam, *Mulinia lateralis* (Say), in Long Island Sound. Veliger.

[22] Santos S., Simon J. (1980). Response of soft-bottom benthos to annual catastrophic disturbance in a south Florida estuary. Mar. Ecol. Prog. Ser..

[23] Yang Z., Huang X., Wang H., Pan H., Wang X., Teng M., Ren Q., Bao Z. (2021). Effects of microalgae diets and stocking density on larval growth, survival and metamorphosis of dwarf surfclam, *Mulinia lateralis*. Aquaculture.

[24] Yang Z., Wang H., Li M., Teng M., Wang X., Zhao A., Huang X., Hu J., Bao Z. (2022). Optimizing Microalgae Diet, Temperature, and Salinity for Dwarf Surf Clam, *Mulinia lateralis*, Spat Culture. Front. Mar. Sci..

[25] Woffelman C. (2004). DNAMAN7.0 for Windows, Lynon Biosoft.

[26] Kumar S., Stecher G., Tamura K. (2015). MEGA7: Molecular evolutionary genetics analysis version 7.0 for bigger datasets. Mol. Biol. Evol..

[27] Surm J.M., Prentis P.J., Pavasovic A. (2015). Comparative analysis and distribution of omega-3 lcPUFA biosynthesis genes in marine molluscs. PLoS ONE.

[28] Surm J.M., Toledo T.M., Prentis P.J., Ana P. (2018). Insights into the phylogenetic and molecular evolutionary histories of Fad and Elovl gene families in Actiniaria. Ecol. Evol..

[29] Ramos-Llorens M., Hontoria F., Navarro J., Ferrier D., Monroig Ó. (2023). Functionally diverse front-end desaturases are widespread in the phylum Annelida. BBA—Mol. Cell Biol. Lipids.

[30] Hastings N., Agaba M., Tocher D.R., Leaver M.J., Dick J.R., Sargent J.R., Teale A.J. (2001). A vertebrate fatty acid desaturase with Δ5 and Δ6 activities. Proc. Natl. Acad. Sci. USA.

[31] Nakamura M.T., Nara T.Y. (2004). Structure, function, and dietary regulation of Δ6, Δ5, and Δ9 desaturases. Annu. Rev. Nutr..

[32] Monroig Ó., Li Y., Tocher D.R. (2011). Delta-8 desaturation activity varies among fatty acyl desaturases of teleost fish: High activity in delta-6 desaturases of marine species. Comp. Biochem. Physiol. B Biochem. Mol. Biol..

[33] Park W.J., Kothapalli K.S., Lawrence P., Tyburczy C., Brenna J.T. (2009). An alternate pathway to long-chain polyunsaturates: The FADS2 gene product Δ8-desaturates 20: 2n-6 and 20: 3n-3. J. Lipid Res..

[34] Cho H.P., Nakamura M.T., Clarke S.D. (1999). Cloning, expression, and nutritional regulation of the mammalian Δ-6 desaturase. J. Biol. Chem..

[35] Marquardt A., Stöhr H., White K., Weber B.H. (2000). cDNA cloning, genomic structure, and chromosomal localization of three members of the human fatty acid desaturase family. Genomics.

[36] Li Y., Monroig O., Zhang L., Wang S., Zheng X., Dick J.R., You C., Tocher D.R. (2010). Vertebrate fatty acyl desaturase with Δ4 activity. Proc. Natl. Acad. Sci. USA.

[37] Monroig Ó., Tocher D.R., Hontoria F., Navarro J.C. (2013). Functional characterisation of a Fads2 fatty acyl desaturase with Δ6/Δ8 activity and an Elovl5 with C16, C18 and C20 elongase activity in the anadromous teleost meagre (*Argyrosomus regius*). Aquaculture.

[38] Zhao R., Yang C.-R., Wang Y.-X., Xu Z.-M., Li S.-Q., Li J.-C., Sun X.-Q., Wang H.-W., Wang Q., Zhang Y. (2023). Fads2b Plays a Dominant Role in ∆6/∆5 Desaturation Activities Compared with Fads2a in Common Carp (*Cyprinus carpio*). Int. J. Mol. Sci..

[39] Monroig Ó., Hontoria F., Varó I., Tocher D.R., Navarro J.C. (2016). Investigating the essential fatty acids in the common cuttlefish *Sepia officinalis* (Mollusca, Cephalopoda): Molecular cloning and functional characterisation of fatty acyl desaturase and elongase. Aquaculture.

[40] Liu H., Guo Z., Zheng H., Wang S., Wang Y., Liu W., Zhang G. (2014). Functional characterization of a Δ5-like fatty acyl desaturase and its expression during early embryogenesis in the noble scallop *Chlamys nobilis Reeve*. Mol. Biol. Rep..

[41] Monroig Ó., Shu-Chien A., Kabeya N., Tocher D.R., Castro L.F.C. (2022). Desaturases and elongases involved in long-chain polyunsaturated fatty acid biosynthesis in aquatic animals: From genes to functions. Prog. Lipid Res..

[42] Monroig Ó., Rotllant J., Sánchez E., Cerdá-Reverter J.M., Tocher D.R. (2009). Expression of long-chain polyunsaturated fatty acid (LC-PUFA) biosynthesis genes during zebrafish *Danio rerio* early embryogenesis. Biochim. Biophys. Acta.

[43] Liu H., Zheng H., Wang S., Wang Y., Li S., Liu W., Zhang G. (2013). Cloning and functional characterization of a polyunsaturated fatty acid elongase in a marine bivalve noble scallop *Chlamys nobilis Reeve*. Aquaculture.

[44] Sarker M.A.-A., Yamamoto Y., Haga Y., Sarker M.S.A., Miwa M., Yoshizaki G., Satoh S. (2011). Influences of low salinity and dietary fatty acids on fatty acid composition and fatty acid desaturase and elongase expression in red sea bream *Pagrus major*. Fish. Sci..

[45] Wu D.L., Huang Y.H., Liu Z.Q., Yu P., Gu P.H., Fan B., Zhao Y.L. (2018). Molecular cloning, tissue expression and regulation of nutrition and temperature on Delta 6 fatty acyl desaturase-like gene in the red claw crayfish (*Cherax quadricarinatus*). Comp. Biochem. Physiol. B Biochem. Mol. Biol..

[46] Lin Z., Hao M., Zhu D., Li S., Wen X. (2017). Molecular cloning, mRNA expression and nutritional regulation of a Δ6 fatty acyl desaturase-like gene of mud crab, *Scylla paramamosain*. Comp. Biochem. Physiol. B Biochem. Mol. Biol..

[47] Li Y.Y., Hu C.B., Zheng Y.J., Xia X.A., Xu W.J., Wang S.Q., Chen W.Z., Sun Z.W., Huang J.H. (2008). The effects of dietary fatty acids on liver fatty acid composition and Delta(6)-desaturase expression differ with ambient salinities in *Siganus canaliculatus*. Comp. Biochem. Physiol. B Biochem. Mol. Biol..

[48] Monroig S., Zheng X., Morais S., Leaver M.J., Taggart J.B., Tocher D.R. (2010). Multiple genes for functional 6 fatty acyl desaturases (Fad) in Atlantic salmon (*Salmo salar* L.): Gene and cDNA characterization, functional expression, tissue distribution and nutritional regulation. BBA—Mol. Cell Biol. Lipids.

[49] Gregory M.K., Collins R.O., Tocher D.R., James M.J., Turchini G.M. (2016). Nutritional regulation of long-chain PUFA biosynthetic genes in rainbow trout (*Oncorhynchus mykiss*). Br. J. Nutr..

[50] Yang C., Hao R., He C., Deng Y., Wang Q. (2022). Cloning and functional characterization of PmΔ5FAD in pearl oyster *Pinctada fucata martensii*. Aquac. Rep..

